# Sleep disturbance and quality of life among university freshmen in Qinghai–Tibet Plateau of China

**DOI:** 10.3389/fpsyt.2022.996996

**Published:** 2022-11-08

**Authors:** Tiantian Zhang, Li Lu, Yan-Ming Ren, Yu-Ying Liu, Kamila Angelika Hynek, Jie Gao, Hong-Ru Chen, Hong-Yi Shen, Xiang-Yun Gai, Zhan-Cui Dang, Shou Liu

**Affiliations:** ^1^Department of Nosocomial Infection Management, Xi’an Central Hospital, College of Medicine, Xi’an Jiaotong University, Xi’an, China; ^2^Health Management and Policy Institute, School of Public Policy and Administration, Xi’an Jiaotong University, Xi’an, China; ^3^Department of Public Health, Medical College, Qinghai University, Xining, China; ^4^Division for Mental and Physical Health, Norwegian Institute of Public Health, Oslo, Norway; ^5^Department of Clinical Medicine, Qinghai Institute of Health Sciences, Xining, China; ^6^Department of Public Education, Xining Urban Vocational & Technical College, Xining, China; ^7^School of Pharmacy, Qinghai Nationalities University, Xining, China

**Keywords:** China, quality of life, Qinghai-Tibet Plateau, sleep disturbance, university freshmen

## Abstract

**Purpose:**

University freshmen are particularly vulnerable as they are undergoing the transition from high school to university with a range of changes. Sleep problems among this group in the Qinghai–Tibet Plateau of China were barely studied. This study aimed to explore sleep disturbance, and its association with quality of life (QoL) and demographic and clinical characteristics among university freshmen in Qinghai–Tibet Plateau, China.

**Methods:**

A multistage stratified cluster random sampling method was performed to recruit student participants with a structured questionnaire to collect sociodemographic and clinical characteristics, and lifestyle behaviors. Sleep disturbance including three aspects of sleep disturbance (i.e., difficulty initiating sleep (DIS), difficulty maintaining sleep (DMS), and early morning awakening (EMA)) was assessed using standardized measurement. Multiple logistic regression models were applied to analyze the data.

**Results:**

Among included 2,769 freshmen, the prevalence of sleep disturbance was 14.8% (95% CI: 14.2–15.5%), and corresponding prevalence of DIS, DMS, and EMA was 8.2% (95% CI: 7.7–8.7%), 8.3% (95% CI: 7.8–8.8%), and 4.2% (95% CI: 3.8–4.6%), respectively. Freshmen with sleeping disturbance had significantly lower QoL in physical [*F*_(1, 2769)_ = 60.23, *p* < 0.001], psychological [*F*_(1, 2769)_ = 46.18, *p* < 0.001], social [*F*_(1, 2769)_ = 23.04, *p* < 0.001], and environment [*F*_(1, 2769)_ = 6.07, *p* = 0.01] domains. Multiple logistic regression analyses revealed that having breakfast five times a week or less (less than three times, OR = 1.79, 95% CI: 1.34–2.40; 3–5 times, OR = 1.40, 95% CI: 1.09–1.79), self-perceived severe Internet dependence (OR = 1.71, 95% CI: 1.11–2.65), self-perceived poor health status (OR = 3.44, 95% CI: 2.06–5.74), high academic stress (OR = 1.42, 95% CI: 1.13–1.78), poor relationship with classmates (OR = 3.44, 95% CI: 1.53–7.71), and severe ADHD symptoms (OR = 1.08, 95% CI: 1.05–1.12) were positively associated with sleeping disturbance.

**Conclusion:**

Sleep disturbance was common among freshmen and is associated with poorer QoL. Prevention and intervention strategies should be developed and implemented, especially among the vulnerable university freshman groups.

## Introduction

Sleep disturbance is one of the most common health problems among university students (1), and the corresponding prevalence varies greatly worldwide. For instance, the prevalence of sleep disturbance among university students ranged between 27.0% in the United States and as high as 70.0% in Iran (2, 3). One meta-analysis including 76 studies revealed that the prevalence of sleep disturbance among Chinese university students was 25.7% (4). Sleep disturbance among students is found to be negatively associated with both mental and physical health, such as academic failure, depression, and poor quality of life (5–7), but also adverse health behaviors, such as Internet addiction, sedentary, and binge-eating behavior (8–10). Individuals with sleep disturbance are characterized by being insufficiently physically active, having poor social relationships and economic status, and reporting greater academic stress (11–14). It was demonstrated that students with unhealthy lifestyle behaviors like cigarette smoking had a high risk of several types of sleep disturbance, such as longer latency to fall asleep and short total sleep duration (15). Previous research found females to be 2.6 times as likely as males to suffer from sleep disturbance (16).

University freshmen are in particularly vulnerable position, as they are undergoing the transition from high school to college or university, with a range of changes including experience lifestyle and social behavior changes (17, 18), for example, being away from home and families, which could help them to casting off the shackles of parents and engage in more social activities (19). It was evidenced that university freshmen are at higher risk of sleep disturbance as compared to senior students (OR = 1.52, 95% CI: 1.17–1.99) (20). Despite the extensive number of studies focusing on sleep disturbance among university students, those specifically paid close attention to the freshmen group were limited.

Sleep disturbances pose great challenges at high altitude (i.e., Qinghai province). Qinghai Province is located in the eastern part of the Qinghai–Tibet Plateau, with 84.1% of the total area above 3,000 m above sea level. Qinghai Province has a unique climatic environment, with multiethnic communities (Han 52.29%, Tibetan 25.23%, Hui 14.78%, Tu 3.55%, and others 4.15% in 2019). There are only few studies on the relationship between sleep and multiethnic group.

We, therefore, conducted this study to explore the prevalence of sleep disturbance among freshmen in Qinghai–Tibet Plateau and compare the quality of life between freshmen with and without sleep disturbance. Then, we examine the association between sleep disturbance and a range of sociodemographic and clinical characteristics. Based on the previous study suggesting the increased risk of sleep disturbance among freshmen on mental health (21), we hypothesized that freshmen university students with sleep disturbances would have a poor quality of life (QoL).

## Materials and methods

### Study design and participants

This cross-sectional study is a school-based mental health survey. It was conducted in December 2019 in Qinghai province, China, which is a plateau region with an average altitude of 2,300 m. A multistage stratified cluster random sampling method was performed to recruit student participants. Four universities (Qinghai University, Qinghai Nationalities University, Qinghai Institute of Health Sciences, and Xining Urban Vocational & Technical College) were selected from twelve universities or colleges in this region based on affiliation levels and classifications. A stratified (majors were taken as the indicator) random sampling method was used to select the classes among the freshmen in each university or college, and cluster sampling was then used in each class. The details concerning the study design have been previously described (22, 23). Those who were fully enrolled were included in this study. Participants with chronic diseases or tumors that can strongly associate with sleep were excluded. The study was approved by the Ethics Committee of the Medical College of Qinghai University, and all participants provided informed consent. This survey was proceeded by following the principles of anonymity and voluntariness.

### Measures

Sociodemographic and clinical characteristics including age, sex (female/male), ethnic Han [yes (The Han represent the majority of China’s people)/no], weight, height, residence areas prior to the enrollment (plateau/non-plateau areas), family economic level (low/general/high), weekly breakfast intake (six–seven times/three–five times/less than three times), self-perceived Internet dependence (no or mild/moderate/severe), academic stress (low/general/high), relationship with classmates, teachers, and families (good/general/poor), and self-perceived health status (good/fair/poor) were collected. Body mass index (BMI) was computed as BMI=weight(kg)/height^2^(m). BMI was divided into four categories according to the modified criteria for the Chinese population, that is, underweight (<18.5 kg/m^2^), normal (18.5–23.9 kg/m^2^), overweight (24.0–27.9 kg/m^2^), and obesity (≥28 kg/m^2^) (24, 25).

Sleep disturbances in the past month were measured by asking the following questions (26): “Do you have difficulties in falling asleep at night (DIS)?”; “Do you have difficulties in maintaining sleep and wake up often (DMS)?”; and “Do you wake up in the midnight or early morning and have difficulties getting back to sleep (EMA)?”. Each question was answered in three options (never, sometimes, and often). The presence of at least one type of sleep disturbance (answer with “often”) was considered as having possible sleep disturbance.

The six-item Adult Attention-Deficit/Hyperactivity Disorder (ADHD) Self-Report Screening Scale for DSM-5 (ASRS-5) was used to measure ADHD symptom. Each item was answered from 0 (never) to 4 (very often). The total score ranges from 0 to 24, and higher total scores indicate severe ADHD (Cronbach’s α = 0.83) (27).

Chinese version of the World Health Organization Quality of Life brief version (WHOQOL-BREF) (28) was used to measure quality of life, which has 26 items and covers physical, psychological, social, and environmental domains. The scores for each domain were transformed on a special 4- to 20-point scale, and higher scores indicate greater QoL (Cronbach’s α = 0.70–0.91) (29).

### Data analyses

All data were imputed into EpiData 3.1 with double-entry method. We excluded participants with 20% or more missing values, and multiple imputation (MI) approach was employed to handle missing data. The Shapiro–Wilk test and Q–Q plot were applied to assess whether continuous variables were normally distributed. The number (*n*) and percentage (%) or the mean and standard deviation (MSD) were used to describe the sociodemographic and clinical characteristics, as appropriate. Differences in sociodemographic and clinical characteristics between students with and without sleep disturbance were compared with the independent t-test, the Mann–Whitney *U*-test, and the chi-square test. Analysis of covariance (ANCOVA) was separately conducted to compare the four domains of QoL between the two groups after controlling the variables with significant differences in univariate analysis. Four multiple logistic analyses were applied to explore the association between potential characteristics and sleeping disturbance and its three aspects, by, respectively, setting sleep disturbance and its three types as dependent variables, and factors with significant difference in univariate analysis (sleep disturbance and its three aspects) as independent variables. We used the Hosmer–Lemeshow test to evaluate the goodness of each model and took advantage of R “*forestplot”* package (30) to draw forest plot to visually display the results. SPSS 25.0 was used to analyze data, and the significant level was set at 0.05 (two-tailed).

## Results

A total of 3,000 questionnaires were sent, among which 2,899 were returned giving a response rate of 96.6%. By excluding participants with 20% or more missing values, data from 2,769 freshmen (age:19.16 ± 1.40, 1,835 female students) were analyzed in the current study. Of these participants, 41.4% are Han ethnic and 58.6% are ethnic minorities. The prevalence of sleep disturbance among freshmen was 14.8% (95% CI: 14.2–15.5%; *n* = 411), and the corresponding prevalence of DIS, DMS, and EMA was 8.2% (95% CI: 7.7–8.7%; *n* = 227), 8.3% (95% CI: 7.8–8.8%; *n* = 229), and 4.2% (95% CI: 3.8–4.6%; *n* = 116), respectively. Of the students with sleep disturbance, 13.1% (54/411) of them reported having taken hypnotics.

The Shapiro–Wilk test showed that age, ADHD score, and QoL in physical, psychological, environment, and social domains were not normally distributed. Table 1 displays the comparisons of sociodemographic and clinical characteristics between freshmen with and without sleep disturbance, DIS, DMS, and EMA. The QoL among freshmen without sleep disturbance was 14.33 ± 2.05, 13.54 ± 2.54, 14.20 ± 2.65, and 13.36 ± 2.39 in physical, psychological, social, and environment domains, respectively. The corresponding index among freshmen with sleep disturbance was 12.93 ± 2.28, 11.88 ± 2.87, 12.98 ± 2.91, and 12.45 ± 2.54.

**TABLE 1:** Sociodemographic characteristics of the participants.

Variables	Categories	Total (*N* = 2,769)	With possible sleep disturbance (*n* = 411)		With DIS (*n* = 227)		With DMS (*n* = 229)		With EMA (*n* = 116)	
						
		*n* (%)	*n* (%)	Statistics^a^	*n* (%)	Statistics^a^	*n* (%)	Statistics^a^	*n* (%)	Statistics^a^
Sex	Female	1,835 (66.3)	279 (67.9)	0.562	140 (61.7)	2.336	168 (73.4)	5.619*	80 (69.0)	0.394
	Male	934 (33.7)	132 (32.1)		87 (38.3)		61 (26.6)		36 (31.0)	
Ethnic han	Yes	1,145 (41.4)	188 (45.7)	3.840	109 (48.0)	4.532*	99 (43.2)	0.364	49 (42.2)	0.040
	No^#^	1,624 (58.6)	223 (54.3)		118 (52.0)		130 (56.8)		67 (57.8)	
BMI (kg/m^2^)	Normal (18.5–23.9)	1,702 (61.5)	233 (56.7)	6.554	125 (55.1)	8.510*	121 (52.8)	7.897*	65 (56.0)	2.798
	Underweight (<18.5)	740 (26.7)	129 (31.4)		72 (31.7)		75 (32.8)		36 (31.0)	
	Overweight (24.0–27.9)	153 (5.5)	20 (4.9)		9 (4.0)		16 (7.0)		5 (4.3)	
	Obesity (≥28)	174 (6.3)	29 (7.1)		21 (9.3)		17 (7.4)		10 (8.6)	
Residence areas prior to the enrolment	Plateau	2,371 (85.6)	353 (85.9)	0.027	191 (84.1)	0.443	203 (88.7)	1.850	93 (80.2)	2.926
	Non-plateau areas	398 (14.4)	58 (14.1)		36 (15.9)		26 (11.4)		23 (19.8)	
Family economic level	High	120 (4.3)	13 (3.2)	13.477**	7 (3.1)	7.588*	10 (4.4)	3.574	6 (5.2)	0.644
	General	2,136 (77.1)	296 (72.0)		163 (71.8)		166 (72.5)		86 (74.1)	
	Low	513 (18.5)	102 (24.8)		57 (25.1)		53 (23.1)		24 (20.7)	
Weekly breakfast	6–7 times	1,404 (50.7)	166 (40.4)	30.539***	88 (38.8)	19.901***	103 (45.0)	12.681**	44 (37.9)	17.848***
	3–5 times	927 (33.5)	147 (35.8)		83 (36.6)		71 (31.0)		38 (32.8)	
	Less than 3 times	438 (15.8)	98 (23.8)		56 (24.7)		55 (24.0)		34 (29.3)	
Self-perceived Internet dependence	No/Mild	1,521 (54.9)	199 (48.4)	27.706***	101 (44.5)	16.271***	112 (48.9)	25.251***	63 (54.3)	6.441*
	Moderate	1,118 (40.4)	173 (42.1)		106 (46.7)		91 (39.7)		42 (36.2)	
	Severe	130 (4.7)	39 (9.5)		20 (8.8)		26 (11.4)		11 (9.5)	
Academic stress	Low	129 (4.7)	15 (3.7)	22.337***	12 (5.3)	10.359**	7 (3.1)	8.579*	5 (4.3)	4.204
	Moderate	1,758 (63.5)	224 (54.5)		122 (53.7)		130 (56.8)		64 (55.2)	
	High	882 (31.9)	172 (41.9)		93 (41.0)		92 (40.2)		47 (40.5)	
Relationship with classmates	Good	1,511 (54.6)	178 (43.3)	41.215***	94 (41.4)	26.352***	106 (46.3)	26.861***	53 (45.7)	3.976
	Fair	1,226 (44.3)	219 (53.3)		125 (55.1)		113 (49.4)		61 (52.6)	
	Poor	32 (1.2)	14 (3.4)		8 (3.5)		10 (4.4)		2 (1.7)	
Relationship with teachers	Good	1,237 (44.7)	160 (38.9)	7.166*	82 (36.1)	7.315*	94 (41.1)	2.869	50 (43.1)	2.870
	Fair	1,489 (53.8)	242 (58.9)		141 (62.1)		129 (56.3)		62 (53.5)	
	Poor	43 (1.6)	9 (2.2)		4 (1.8)		6 (2.6)		4 (3.5)	
Relationship with families	Good	2,429 (87.7)	332 (80.8)	22.082***	176 (77.5)	23.865***	183 (79.9)	15.621***	87 (75.0)	18.209***
	Fair	315 (11.4)	72 (17.5)		47 (20.7)		41 (17.9)		27 (23.3)	
	Poor	25 (0.9)	7 (1.7)		4 (1.8)		5 (2.2)		2 (1.7)	
Self-perceived health status	Good	1,388 (50.1)	131 (31.9)	82.368***	74 (32.6)	42.212***	68 (29.7)	65.124***	23 (19.8)	70.656***
	Fair	1,295 (46.8)	249 (60.6)		135 (59.5)		139 (60.7)		78 (67.2)	
	Poor	86 (3.1)	31 (7.5)		18 (7.9)		22 (9.6)		15 (12.9)	

		**Mean ± SD**	**Mean ± SD**	**Statistics^b^**	**Mean ± SD**	**Statistics**	**Mean ± SD**	**Statistics^b^**	**Mean ± SD**	**Statistics^b^**

Age (years)		19.16 ± 1.40	19.17 ± 1.28	0.310	19.17 ± 1.33	0.668	19.22 ± 1.29	0.561	19.24 ± 1.40	0.019
ADHD		5.53 ± 3.43	6.80 ± 3.89	7.250***	6.73 ± 3.81	5.122***	7.28 ± 3.92	7.270***	6.74 ± 4.00	3.181**
Physical QoL		14.12 ± 2.14	12.93 ± 2.28	11.803***	12.80 ± 2.24	9.747***	12.91 ± 2.31	8.270***	12.73 ± 2.17	6.897***
Psychological QoL		13.29 ± 2.66	11.88 ± 2.87	10.729***	11.78 ± 2.95	8.218***	11.66 ± 2.92	8.748***	11.47 ± 2.71	7.246***
Social QoL		14.02 ± 2.72	12.98 ± 2.91	7.984***	12.70 ± 3.10	7.039***	12.85 ± 2.88	6.352***	12.76 ± 2.85	4.659***
Environment QoL		13.23 ± 2.43	12.45 ± 2.54	6.188***	12.41 ± 2.52	4.852***	12.39 ± 2.67	4.562***	12.47 ± 2.38	2.865**

ADHD, attention-deficit/hyperactivity disorder; BMI, body mass index; QoL, quality of life; DIS, Difficulties in initiating sleep; DMS, difficulties in maintaining sleep; EAM, early morning awakening. *p < 0.05, **p < 0.01, ***p < 0.001 (two-tailed). ^a^Chi-square test; ^b^Mann–Whitney U-test. ^#^Ethnic minorities include the Tibetan, Hui, and Tu.

ANCOVA analyses showed that, after controlling for BMI, family economic level, weekly breakfast, self-perceived Internet dependence, academic stress, ADHD, and interpersonal relationships (relationship with classmates, teachers, and families), participants with sleep disturbance had significantly lower QoL as compared to those without sleep disturbance in physical domain [*F*_(1, 2769)_ = 60.23, *p* < 0.001], psychological domain [*F*_(1, 2769)_ = 46.18, *p* < 0.001], social domain [*F*_(1, 2769)_ = 23.04, *p* < 0.001], and environment domain [*F*_(1, 2769)_ = 6.07, *p* = 0.01].

**FIGURE 1:**
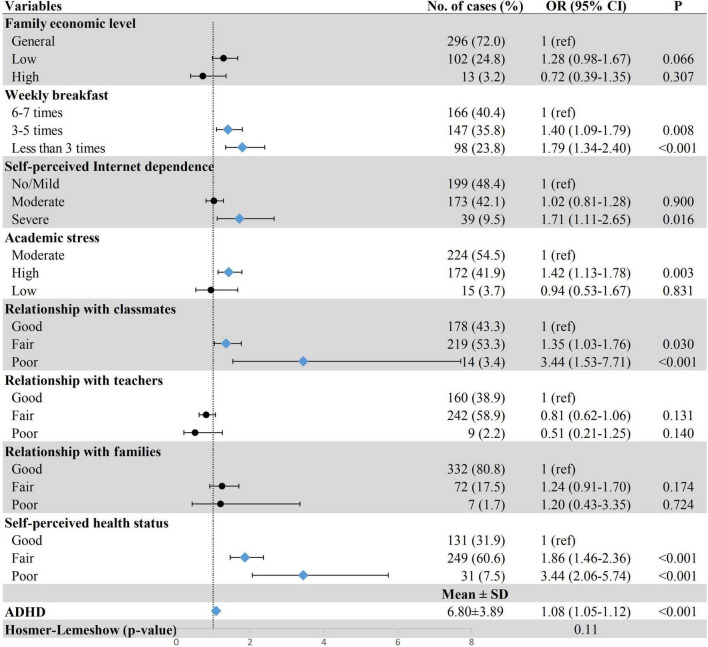
Association between demographic and clinical characteristics and possible sleep disturbance. Row with blue diamond indicates statistical significance. ADHD, attention-deficit/hyperactivity disorder; OR, odds ratio; 95% CI, 95% confidence interval.

Multiple logistic regression showed that having breakfast five times a week or less (less than three times, OR = 1.79, 95% CI: 1.34–2.40; three–five times, OR = 1.40, 95% CI: 1.09–1.79), a self-perceived severe Internet dependence (OR = 1.71, 95% CI: 1.11–2.65), high academic stress (OR = 1.42, 95% CI: 1.13–1.78), poor relationship with classmates (OR = 3.44, 95% CI: 1.53–7.71), self-perceived poor health status (OR = 3.44, 95% CI: 2.06–5.74), and severe ADHD symptoms (OR = 1.08, 95% CI: 1.05–1.12) were positively associated with sleeping disturbance (Figure 1).

**FIGURE 2:**
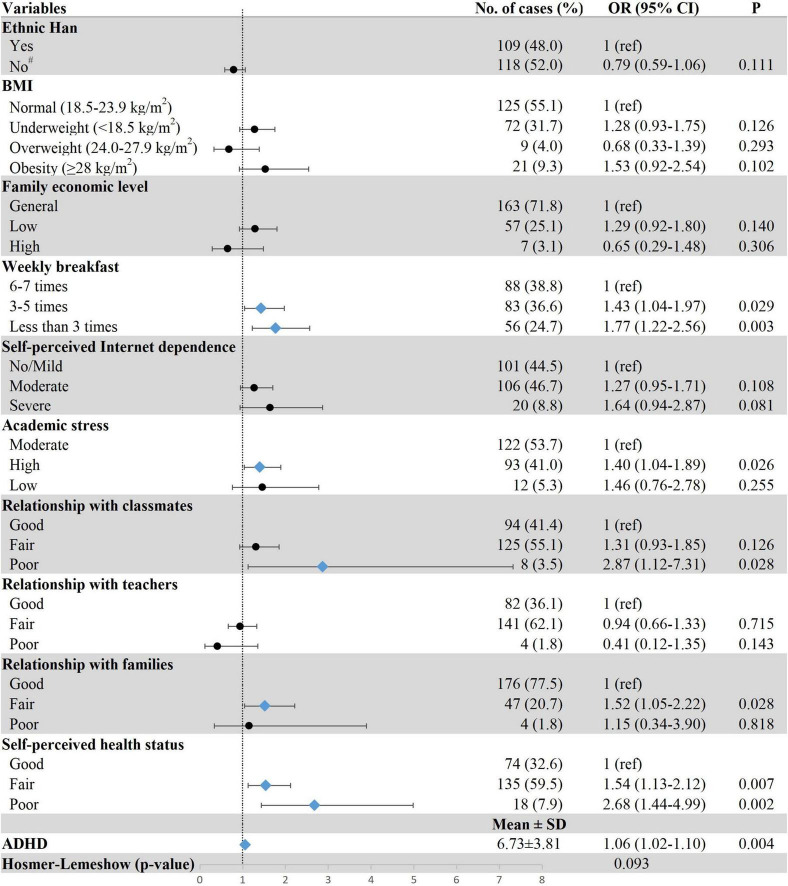
Associations between demographic and clinical characteristics and DIS. Row with blue diamond indicates statistical significance. ^#^Ethnic minorities including the Tibetan, Hui, and Tu. ADHD, attention-deficit/hyperactivity disorder; BMI, body mass index; DIS, difficulties initiating sleep; OR, odds ratio; 95% CI, 95% confidence interval.

**FIGURE 3:**
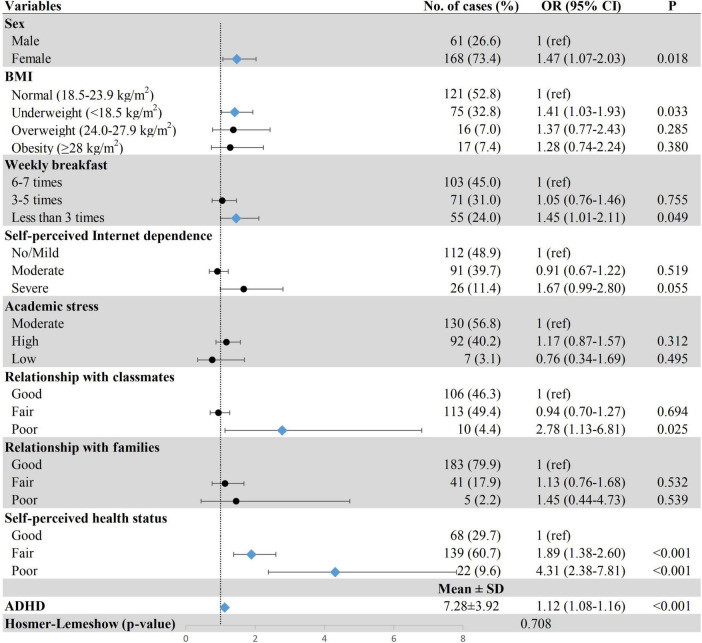
Associations between demographic and clinical characteristics and DMS. Row with blue diamond indicates statistical significance. ADHD, attention-deficit/hyperactivity disorder; BMI, body mass index; DMS, difficulties in maintaining sleep; OR, odds ratio; 95% CI, 95% confidence interval.

**FIGURE 4:**
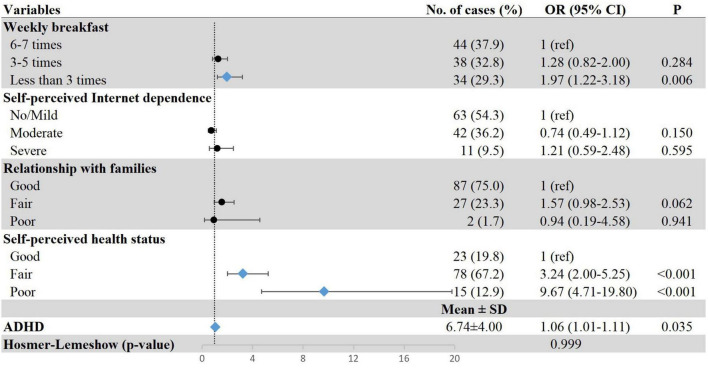
Associations between demographic and clinical characteristics and EMA. Row with blue diamond indicates statistical significance. ADHD, attention-deficit/hyperactivity disorder; EMA, early-morning awakening with inability to return to sleep; OR, odds ratio; 95% CI, 95% confidence interval.

Figures 2–4 show the results of multiple logistic regression analyses for the three patterns of sleep disturbances. Students having breakfast less than three times a week (DIS, OR = 1.77, 95% CI: 1.22–2.56; DMS, OR = 1.45, 95% CI: 1.01–2.11; EMA, OR = 1.97, 95% CI: 1.22–3.18), with self-perceived poor health status (DIS, OR = 2.68, 95% CI: 1.44–4.99; DMS, OR = 4.31, 95% CI: 2.38–7.81; EMA, OR = 9.67, 95% CI: 4.71–19.80), and severe ADHD symptoms (DIS, OR = 1.06, 95% CI: 1.02–1.10; DMS, OR = 1.12, 95% CI: 1.08–1.16; EMA, OR = 1.06, 95% CI: 1.01–1.11) were more likely to have different types of sleep disturbance. Poor relationship with classmates was positively associated with DIS (OR = 2.87, 95% CI: 1.12–7.31) and DMS (OR = 2.78, 95% CI: 1.13–6.81); yet due to a small number of individuals in these groups, the results should be interpreted with caution. The results also showed that freshmen with high academic stress (OR = 1.40, 95% CI: 1.04–1.89) were susceptible to DIS. Female freshmen (OR = 1.47, 95% CI: 1.07–2.03) and underweight students (OR = 1.41, 95% CI: 1.03–1.93) were more prone to have DMS.

## Discussion

We found that the prevalence of sleep disturbance among freshmen in Qinghai–Tibet plateau was 14.8%, which was higher than the corresponding global figure of 10.4% measured in 20,222 undergraduate students from 26 countries (31). However, it was lower than the figure among freshmen in the United States (42.0%) (32), Saudi Arabia (75.4%) (33), Japan (33.3%) (21), and another study of China (37.9%) (34). The discrepancies could be explained by the following aspects. First, freshmen were more likely to suffer from sleep disturbance given the fact that they have fewer experiences than students in other grades in dealing with new pattern of life and academic pressure (20). It should also be noticed that the study was performed in December, and the academic pressure could also have partially explained the effects of final examination. Second, sleep is associated with varying socioeconomic factors. For example, people living in countries with high economic pressure and poor healthcare are susceptible to sleep disturbance (35). Third, racial differences in sleep quality were common (36), even within the same race (37). In addition, the definition, assessment tools, and investigative method used are not consistent across studies. Therefore, the direct comparisons of these rates should be made with caution.

As hypothesized, freshmen with sleep disturbance reported poorer QoL in physical, psychological, social, and environmental domains than those without sleep disturbance. Furthermore, we found breakfast consumption less than five times a week being positively associated with sleep disturbance. Skipping breakfast is relatively common among university students, with a range of 40.0% to 70.0% (38, 39). A cross-sectional study showed that having breakfast five or more days a week is a probable protective factor of sleep (40). Except for circadian rhythm, the association of breakfast intake and sleep disturbance was also related to a lack of daytime routine (41, 42). The association between Internet dependence and sleep disturbance was also reported among Japanese adolescents (43) and another large-scale study among Chinese adolescents (44). Another study found using the Internet as an unstructured activity with no fixed start and ending points can take up sleep time and cause sleep disturbance (45, 46). Another possible mechanism is that the screen light of Internet-using devices inhibits the secretion of melatonin, which will reduce subjective and objective sleep signals resulting in syndrome sleep phase delay (47, 48).

According to previous studies, negative relationship with family, teacher, or classmate may produce harmful emotion and behavior (49, 50), which could further lead to sleep disturbance. In our study, only poor relationship with classmates was significantly associated with sleep disturbance. The relationship with classmates is one of the most essential interpersonal contact when freshmen involve in university life. Studies have proven that students’ harmonious interpersonal relationships would benefit their sleep (51, 52); students with high-level interpersonal relationships experience less interpersonal pressure and mood swings which is propitious to sleep (53). Notably, fair relationship with families was associated with DIS. Previous findings suggested that under the context of outside pressure, cohesive relationship with families serves as a “safety signal” and may provide sense of stability which is necessary for healthy sleep (54). However, we found no association between poor relationship with families and DIS as only 25 participants had poor relationship with families which is less than one % of the total sample size.

We also found that about half of the students in this study reported good self-perceived health status and there is a strong independent association between self-perceived health status and sleep disturbance, which was consistent with the previous study (55). In this sample, the odds of suffering from sleep disturbance among freshmen with ADHD are higher than those who are not having ADHD. The previous study indicated that the odds of experiencing sleep disturbance for adolescents with ADHD were more than six times of those without (56). A meta-analysis revealed that youth have worse sleep quality after taking stimulants (57), and the psychostimulant use among participants with higher ADHD symptoms was not assessed in this study. Besides, it has been reported that untreated ADHD patients had melatonin onset delay at night which was related with circadian rhythm disorder (58).

Deviation from normal weight (BMI = 18.5–24.9) is found to be significantly associated with sleep disturbance in epidemiological studies conducted in European countries (59, 60). We only found association between underweight and DIS. The prevalence of underweight was obviously higher than the prevalence of obesity and overweight in our study. In addition, females were more likely to develop DIS, which is consistent with prior research indicating that females have 50% higher risk to develop DIS than males (61). These sex differences may be partially explained by changes in hormones during menstruation among females, which increase the risk of sleep disturbance (62).

High-altitude environments were also found to have an adverse effect on the physiological function of people who are used to living at low altitude (63). However, we did not ascertain the significant association between residence areas prior to the enrollment (plateau or non-plateau areas) and sleep disturbance. This may be related to the fact that this investigation was conducted three months after the start of academic year and they may have adapted to the plateau environment. It should be noted that Qinghai province is a plateau region with an average altitude of 2,300 m, but previous surveys revealed that stress responses to high altitude are usually limited to over 3,000 m above sea level (64, 65).

Our study results suggested that university freshmen suffered from sleep disturbance, which was associated with poor QoL. School-based preventions and interventions should be considered and implemented to prevent sleep disturbance and hence the reduced QoL of those potentially affected (66). For instance, psychological counseling services through face-to-face care and telehealth should be both adopted. Helping the establishment of exceptional self-regulation in freshmen also contributes to a series of positive health consequences with long-term implications (67).

However, the results of this study should be interpreted considering several limitations. The first one related to the cross-sectional study design and self-report data failed to help us report any causal relationship between sleep disturbance and related factors. Second, the measurement of independent variables, such as self-perceived Internet dependence, which is single item measures and may lower the reliability and validity. Third, this study intends to research the sleep disturbance of freshmen in Qinghai–Tibet plateau, but only Qinghai province is included. Further studies should include more provinces in Qinghai–Tibet plateau to validate the results. However, our study benefited greatly from the sample size that ensured the statistical power and a detailed assessment of sleep disturbance including three dimensions (i.e., DIS, DMS, and EMA).

## Conclusion

This study highlighted that sleep disturbance is common among university freshmen in Qinghai–Tibet plateau and is associated with poorer QoL of those affected. Students with poor relationship with classmates, irregularly breakfast intake, self-perceived Internet dependence, self-perceived poor health status, high academic stress, and ADHD symptom were more likely to have sleep disturbance. The focus on development and implementation of preventive measures targeted toward freshmen and their sleep problems could not only promote healthy sleep but also improve students’ academic performance. Improving consciousness of freshmen about the modifiable lifestyle factors having adverse effect on their sleep is also of great importance.

## Data availability statement

The raw data supporting the conclusions of this article will be made available by the authors, without undue reservation.

## Ethics statement

This study was approved and reviewed by the Ethics Committee of the Medical College of Qinghai University. Written informed consent to participate was obtained from all participants.

## Author contributions

TZ, SL, and LL: study design. TZ, LL, Y-YL, JG, H-RC, H-YS, X-YG, Y-MR, and Z-CD: data collection, analysis, and interpretation. TZ, LL, SL, and KH: drafting of the manuscript. All authors approved the final version for publication.

## References

[1] Putilov AA Sveshnikov DS Bakaeva ZB Yakunina EB Starshinov YP Torshin VI Differences between male and female university students in sleepiness, weekday sleep loss, and weekend sleep duration. *J Adolesc.* (2021) 88:84–96. 10.1016/j.adolescence.2021.02.006 33667792

[2] Becker SP Jarrett MA Luebbe AM Garner AA Burns GL Kofler MJ. Sleep in a large, multi-university sample of college students: sleep problem prevalence, sex differences, and mental health correlates. *Sleep Health.* (2018) 4:174–81. 10.1016/j.sleh.2018.01.001 29555131 PMC5863586

[3] Sarbazvatan H Amini A Aminisani N Shamshirgaran SM. Sleep quality and academic progression among students of tabriz university of medical sciences, northwest of iran. *Res Dev Med Educ.* (2017) 6:29–33. 10.15171/rdme.2017.006

[4] Li L Wang YY Wang SB Zhang L Li L Xu DD Prevalence of sleep disturbances in Chinese university students: a comprehensive meta-analysis. *J Sleep Res.* (2018) 27:e12648. 10.1111/jsr.12648 29383787

[5] Titova OE Hogenkamp PS Jacobsson JA Feldman I Schioth HB Benedict C. Associations of self-reported sleep disturbance and duration with academic failure in community-dwelling Swedish adolescents: sleep and academic performance at school. *Sleep Med.* (2015) 16:87–93. 10.1016/j.sleep.2014.09.004 25441744

[6] Matsui K Kuriyama K Yoshiike T Nagao K Ayabe N Komada Y The effect of short or long sleep duration on quality of life and depression: an internet-based survey in Japan. *Sleep Med.* (2020) 76:80–5. 10.1016/j.sleep.2020.10.012 33120132

[7] Xu YM Pu SS Li Y Zhong BL. Possible avoidant personality disorder magnifies the association between bullying victimization and depressive symptoms among chinese university freshmen. *Front Psychiatry.* (2022) 13:822185. 10.3389/fpsyt.2022.822185 35250671 PMC8891554

[8] Chen YL Gau SS. Sleep problems and internet addiction among children and adolescents: a longitudinal study. *J Sleep Res.* (2016) 25:458–65. 10.1111/jsr.12388 26854132

[9] Yang Y Shin JC Li D An R. Sedentary behavior and sleep problems: A systematic review and meta-analysis. *Int J Behav Med.* (2017) 24:481–92. 10.1007/s12529-016-9609-0 27830446

[10] Allison KC Spaeth A Hopkins CM. Sleep and eating disorders. *Curr Psychiatry Rep.* (2016) 18:92. 10.1007/s11920-016-0728-8 27553980

[11] Kovacevic A Mavros Y Heisz JJ Fiatarone Singh MA. The effect of resistance exercise on sleep: A systematic review of randomized controlled trials. *Sleep Med Rev.* (2018) 39:52–68. 10.1016/j.smrv.2017.07.002 28919335

[12] Gordon AM Carrillo B Barnes CM. Sleep and social relationships in healthy populations: A systematic review. *Sleep Med Rev.* (2021) 57:101428. 10.1016/j.smrv.2021.101428 33596514

[13] Zhang W Ohira T Maeda M Nakano H Iwasa H Yasumura S The association between self-reported sleep dissatisfaction after the Great East Japan Earthquake, and a deteriorated socioeconomic status in the evacuation area: the Fukushima Health Management Survey. *Sleep Med.* (2020) 68:63–70. 10.1016/j.sleep.2019.09.004 32028228

[14] Liu B Gao F Zhang J Zhou H Sun N Li L Sleep quality of students from elementary school to university: A cross-sectional study. *Nat Sci Sleep.* (2020) 12:855–64. 10.2147/NSS.S266493 33154689 PMC7605933

[15] Costa M Esteves M. Cigarette smoking and sleep disturbance. *Addict Disord Their Treatment.* (2018) 17:40–8. 10.1097/ADT.0000000000000123

[16] Ahmed AE Al-Jahdali H Fatani A Al-Rouqi K Al-Jahdali F Al-Harbi A The effects of age and gender on the prevalence of insomnia in a sample of the Saudi population. *Ethn Health.* (2017) 22:285–94. 10.1080/13557858.2016.1244624 27846729

[17] Kim M Cardinal BJ. Psychological state and behavioural profiles of freshman enrolled in college and university instructional physical activity programmes under different policy conditions. *Montenegrin J Sports Sci Med.* (2019) 8:13–20. 10.26773/mjssm.190902

[18] Zhang CL Xu YM Zhong BL. The association between smoking and loneliness among Chinese university freshmen. *Ann Transl Med.* (2020) 8:649. 10.21037/atm-20-3523 32566586 PMC7290622

[19] Griggs S Crawford SL. Differences in hope, core self-evaluations, emotional well-being, and health risk behaviors in freshman university students. *Nurs Forum.* (2019) 54:505–12. 10.1111/nuf.12364 31309592 PMC6856360

[20] Li Y Bai W Zhu B Duan R Yu X Xu W Prevalence and correlates of poor sleep quality among college students: a cross-sectional survey. *Health Qual Life Outcomes.* (2020) 18:210. 10.1186/s12955-020-01465-2 32611434 PMC7329462

[21] Supartini A Honda T Basri NA Haeuchi Y Chen S Ichimiya A The impact of sleep timing, sleep duration, and sleep quality on depressive symptoms and suicidal ideation amongst japanese freshmen: The EQUSITE Study. *Sleep Disord.* (2016) 2016:8737654. 10.1155/2016/8737654 27042358 PMC4794596

[22] Lu L Jian S Dong M Gao J Zhang T Chen X Childhood trauma and suicidal ideation among Chinese university students: the mediating effect of Internet addiction and school bullying victimisation. *Epidemiol Psychiatr Sci.* (2020) 29:e152. 10.1017/S2045796020000682 32772993 PMC7443799

[23] Lu L Dong M Jian S Gao J Ye L Chen HR Sex differences in the factors associated with sleep duration in university students: A cross-sectional study. *J Affect Disord.* (2021) 290:345–52. 10.1016/j.jad.2021.04.025 34049087

[24] Cai J Ma A Wang Q Han X Zhao S Wang Y Association between body mass index and diabetes mellitus in tuberculosis patients in China: a community based cross-sectional study. *BMC Public Health.* (2017) 17:228. 10.1186/s12889-017-4101-6 28245792 PMC5331649

[25] He W Li Q Yang M Jiao J Ma X Zhou Y Lower BMI cutoffs to define overweight and obesity in China. *Obesity (Silver Spring).* (2015) 23:684–91. 10.1002/oby.20995 25645003

[26] Liu S Chow IHI Lu L Ren YM Yang HL Jian SY Comparison of sleep disturbances between older nursing home residents in high- and low-altitude areas. *J Geriatr Psychiatry Neurol.* (2020) 33:370–6. 10.1177/0891988719892335 31838930

[27] Ustun B Adler LA Rudin C Faraone SV Spencer TJ Berglund P The world health organization adult attention-deficit/hyperactivity disorder self-report screening scale for DSM-5. *JAMA Psychiatry.* (2017) 74:520–7. 10.1001/jamapsychiatry.2017.0298 28384801 PMC5470397

[28] Felce D Perry J. Quality of life: Its definition and measurement. *Res Dev Disabil.* (1995) 16:51–74. 10.1016/0891-4222(94)00028-8 7701092

[29] Lin CY Lee TY Sun ZJ Yang YC Wu JS Ou HT. Development of diabetes-specific quality of life module to be in conjunction with the World Health Organization quality of life scale brief version (WHOQOL-BREF). *Health Qual Life Outcomes.* (2017) 15:167. 10.1186/s12955-017-0744-3 28835235 PMC5569515

[30] Gordon M Lumley T. *Gordon MMJAfpuggTCRAN.* Vienna: Package ‘forestplot’ (2019).

[31] Peltzer K Pengpid S. Nocturnal sleep problems among university students from 26 countries. *Sleep Breath.* (2015) 19:499–508. 10.1007/s11325-014-1036-3 25017741

[32] Chen W-L Chen J-H. Consequences of inadequate sleep during the college years: Sleep deprivation, grade point average, and college graduation. *Prev Med.* (2019) 124:23–8. 10.1016/j.ypmed.2019.04.017 31034864

[33] Aldhawyan AF Alfaraj AA Elyahia SA Alshehri SZ Alghamdi AA. Determinants of subjective poor sleep quality in social media users among freshman college students. *Nat Sci Sleep.* (2020) 12:279–88. 10.2147/NSS.S243411 32523388 PMC7237109

[34] Ma C Zhou L Xu W Ma S Wang Y. Associations of physical activity and screen time with suboptimal health status and sleep quality among Chinese college freshmen: A cross-sectional study. *PLoS One.* (2020) 15:e0239429. 10.1371/journal.pone.0239429 32946516 PMC7500622

[35] Chen WC Chen SJ Zhong BL. Sense of alienation and its associations with depressive symptoms and poor sleep quality in older adults who experienced the lockdown in wuhan, china, during the COVID-19 Pandemic. *J Geriatr Psychiatry Neurol.* (2022) 35:215–22. 10.1177/08919887221078564 35130783 PMC8899829

[36] George KM Peterson RL Gilsanz P Mungas DM Glymour MM Mayeda ER Racial/Ethnic differences in sleep quality among older adults: kaiser healthy aging and diverse life experiences (KHANDLE) Study. *Ethn Dis.* (2020) 30:469–78. 10.18865/ed.30.3.469 32742152 PMC7360172

[37] Takahashi M Wang G Adachi M Jiang F Jiang Y Saito M Differences in sleep problems between Japanese and Chinese preschoolers: a cross-cultural comparison within the Asian region. *Sleep Med.* (2018) 48:42–8. 10.1016/j.sleep.2017.11.1145 29857290

[38] Mansouri M Sharifi F Shokri A Varmaghani M Yaghubi H Moghadas-Tabrizi Y Breakfast consumption is inversely associated with primary headaches in university students: The MEPHASOUS study. *Complement Ther Med.* (2021) 57:102663. 10.1016/j.ctim.2021.102663 33460743

[39] Ojieabu CE Sholeye O Ojieabu W Onabajo B. Breakfast consumption and associated factors among students in olabisi onabanjo university, ogun state, nigeria. *Value in Health.* (2017) 20:A683–4. 10.1016/j.jval.2017.08.1719

[40] Gomez-Chiappe N Lara-Monsalve PA Gomez AM Gómez DC González JC González L Poor sleep quality and associated factors in university students in Bogota D.C., Colombia. *Sleep Sci.* (2020) 13:125–30. 10.5935/1984-0063.20190141 32742583 PMC7384535

[41] Tambalis KD Panagiotakos DB Psarra G Sidossis LS. Insufficient sleep duration is associated with dietary habits, screen time, and obesity in children. *J Clin Sleep Med.* (2018) 14:1689–96. 10.5664/jcsm.7374 30353810 PMC6175799

[42] Villa-Gonzalez E Huertas-Delgado FJ Chillon P Ramirez-Velez R Barranco-Ruiz Y. Associations between active commuting to school, sleep duration, and breakfast consumption in Ecuadorian young people. *BMC Public Health.* (2019) 19:85. 10.1186/s12889-019-6434-9 30658708 PMC6339393

[43] Tokiya M Itani O Otsuka Y Kaneita Y. Relationship between internet addiction and sleep disturbance in high school students: a cross-sectional study. *BMC Pediatr.* (2020) 20:379. 10.1186/s12887-020-02275-7 32782022 PMC7418409

[44] Guo L Luo M Wang WX Huang GL Xu Y Gao X Association between problematic Internet use, sleep disturbance, and suicidal behavior in Chinese adolescents. *J Behav Addict.* (2018) 7:965–75. 10.1556/2006.7.2018.115 30474380 PMC6376369

[45] Yang J Guo Y Du X Jiang Y Wang W Xiao D Association between Problematic Internet Use and Sleep Disturbance among Adolescents: The Role of the Child’s Sex. *Int J Environ Res Public Health.* (2018) 15:2682. 10.3390/ijerph15122682 30487425 PMC6313705

[46] Luo W Zhong BL Chiu HF. Prevalence of depressive symptoms among Chinese university students amid the COVID-19 pandemic: a systematic review and meta-analysis. *Epidemiol Psychiatr Sci.* (2021) 30:e31. 10.1017/S2045796021000202 33766163 PMC8047400

[47] Petit A Karila L Estellat C Moisan D Reynaud M D’Ortho MP Sleep disorders in Internet addiction. *Presse Med.* (2016) 45(12 Pt 1):1170–7. 10.1016/j.lpm.2016.04.025 27887821

[48] Royant-Parola S Londe V Trehout S Hartley S. The use of social media modifies teenagers’ sleep-related behavior. *Encephale.* (2018) 44:321–8. 10.1016/j.encep.2017.03.009 28602529

[49] Bannon SM Greenberg J Goldson J O’Leary D Vranceanu AMA. Social Blow: The role of interpersonal relationships in mild traumatic brain injury. *Psychosomatics.* (2020) 61:518–26. 10.1016/j.psym.2020.04.003 32408992

[50] Dietz LJ. Family-Based interpersonal psychotherapy: An intervention for preadolescent depression. *Am J Psychother.* (2020) 73:22–8. 10.1176/appi.psychotherapy.20190028 32050785

[51] Jin Y Ding Z Fei Y Jin W Liu H Chen Z Social relationships play a role in sleep status in Chinese undergraduate students. *Psychiatry Res.* (2014) 220:631–8. 10.1016/j.psychres.2014.08.029 25200188

[52] Wang PY Lin PH Lin CY Yang SY Chen KL. Does interpersonal interaction really improve emotion, sleep quality, and self-efficacy among junior college students? *Int J Environ Res Public Health.* (2020) 17:4542. 10.3390/ijerph17124542 32599755 PMC7345085

[53] Gunn HE Troxel WM Hall MH Buysse DJ. Interpersonal distress is associated with sleep and arousal in insomnia and good sleepers. *J Psychosom Res.* (2014) 76:242–8. 10.1016/j.jpsychores.2013.11.010 24529045 PMC4018775

[54] Tsai KM Dahl RE Irwin MR Bower JE McCreath H Seeman TE The roles of parental support and family stress in adolescent sleep. *Child Dev.* (2018) 89:1577–88. 10.1111/cdev.12917 28777438 PMC5814359

[55] Mangrio E Zdravkovic S Sjogren Forss K. The association between self-perceived health and sleep-quality and anxiety among newly arrived refugees in sweden: A quantitative study. *J Immigr Minor Health.* (2020) 22:82–6. 10.1007/s10903-019-00871-z 30788680 PMC6952325

[56] Becker SP Langberg JM Eadeh HM Isaacson PA Bourchtein E. Sleep and daytime sleepiness in adolescents with and without ADHD: differences across ratings, daily diary, and actigraphy. *J Child Psychol Psychiatry.* (2019) 60:1021–31. 10.1111/jcpp.13061 31032953 PMC6692210

[57] Kidwell KM Van Dyk TR Lundahl A Nelson TD. Stimulant medications and sleep for youth with ADHD: A Meta-analysis. *Pediatrics.* (2015) 136:1144–53. 10.1542/peds.2015-1708 26598454

[58] Konofal E Lecendreux M Cortese S. Sleep and ADHD. *Sleep Med.* (2010) 11:652–8. 10.1016/j.sleep.2010.02.012 20620109

[59] Mullane N Bradley C. An investigation into the association between demographic and morbidity factors, and sleep disturbance. *Ir J Med Sci.* (2018) 187:163–75. 10.1007/s11845-017-1640-x 28646468

[60] Sivertsen B Pallesen S Sand L Hysing M. Sleep and body mass index in adolescence: results from a large population-based study of Norwegian adolescents aged 16 to 19 years. *BMC Pediatr.* (2014) 14:204. 10.1186/1471-2431-14-204 25128481 PMC4148405

[61] Zhang B Wing YK. Sex differences in insomnia: a meta-analysis. *Sleep.* (2006) 29:85–93. 10.1093/sleep/29.1.85 16453985

[62] Pengo MF Won CH Bourjeily G. Sleep in women across the life Span. *Chest.* (2018) 154:196–206. 10.1016/j.chest.2018.04.005 29679598 PMC6045782

[63] Kabel AM Al Thumali AM Aldowiala KA Habib RD Aljuaid SS Alharthi HA. Sleep disorders in adolescents and young adults: Insights into types, relationship to obesity and high altitude and possible lines of management. *Diabetes Metab Syndr.* (2018) 12:777–81. 10.1016/j.dsx.2018.04.029 29673929

[64] Hill CM Bucks RS Cellini N Motamedi S Carroll A Heathcote K Cardiac autonomic activity during sleep in high-altitude resident children compared with lowland residents. *Sleep.* (2018) 41:181. 10.1093/sleep/zsy181 30219885

[65] Bian SZ Zhang L Jin J Zhang J-H Li Q-N Chen J-F The onset of sleep disturbances and their associations with anxiety after acute high-altitude exposure at 3700 m. *Transl Psychiatry.* (2019) 9:175. 10.1038/s41398-019-0510-x 31332159 PMC6646382

[66] Li HM Xu YM Zhong BL. Relationship between childhood left-behind experience and quality of life among chinese university freshmen: place of origin matters. *Front Psychiatry.* (2021) 12:789622. 10.3389/fpsyt.2021.789622 34899441 PMC8651710

[67] Friedrich A Schlarb AA. Let’s talk about sleep: a systematic review of psychological interventions to improve sleep in college students. *J Sleep Res.* (2018) 27:4–22. 10.1111/jsr.12568 28618185

